# Optimizing Lymph Node Staging in Non-Small Cell Lung Cancer Surgery: Evidence, Guidelines, and Quality Improvement Strategies

**DOI:** 10.3390/jcm15020831

**Published:** 2026-01-20

**Authors:** Dimitrios E. Magouliotis, Vasiliki Androutsopoulou, Ugo Cioffi, Fabrizio Minervini, Noah Sicouri, Andrew Xanthopoulos, Marco Scarci

**Affiliations:** 1 Department of Cardiac Surgery Research, Lankenau Institute for Medical Research, Wynnewood, PA 19096, USA; magouliotisd@mlhs.org; 2 Department of Cardiothoracic Surgery, University of Thessaly, Biopolis, 41110 Larissa, Greece; androutsopoulouvasiliki@uth.gr; 3 Department of Surgery, University of Milan, 20157 Milan, Italy; ugo.cioffi@guest.unimi.it; 4 Department of Thoracic Surgery, Luzern Kanton Hospital, 6000 Luzern, Switzerland; fabriziominervini@hotmail.com; 5 Department of Neuroscience Pittsburgh Campus, University of Pittsburgh, Pittsburgh, PA 15260, USA; nps67@pitt.edu; 6 Department of Cardiology, University of Thessaly, Biopolis, 41110 Larissa, Greece; andrewvxanth@gmail.com; 7 Department of Cardiothoracic Surgery, Hammersmith Hospital, Imperial College Healthcare, National Health Service (NHS) Trust, London W2 1NY, UK

**Keywords:** lung cancer, non-small-cell lung cancer (NSCLC), surgery, lymph node staging, mediastinal lymphadenectomy, quality improvement

## Abstract

Lymph node evaluation is a central determinant of oncologic quality in the surgical management of non-small-cell lung cancer (NSCLC). Accurate assessment of hilar and mediastinal lymph nodes underpins pathologic staging, informs postoperative treatment decisions, and remains essential for prognostic stratification and assessment of resection completeness. Although international guidelines provide clear recommendations, real-world data consistently demonstrate substantial variability in lymph node staging practices, with inadequate evaluation frequently observed across institutions and surgical settings. Insufficient nodal assessment, manifested as the omission of mediastinal staging, limited station sampling, or low lymph node yield, is associated with reduced nodal upstaging, inappropriate omission of adjuvant therapy, higher recurrence rates, and inferior long-term survival. Contemporary guidance from major societies, including the National Comprehensive Cancer Network, European Society of Thoracic Surgeons, International Association for the Study of Lung Cancer, and the Commission on Cancer, has increasingly converged on a station-based definition of adequacy, emphasizing systematic evaluation of both N1 and N2 nodal stations rather than reliance on absolute node counts alone. In parallel, preoperative mediastinal staging algorithms have evolved toward routine use of endobronchial and esophageal ultrasound as first-line invasive modalities, reserving surgical mediastinoscopy for selected high-risk or inconclusive cases. Evidence from randomized trials, population-level databases, and meta-analyses indicates that thorough nodal assessment improves staging accuracy and survival, while recent data support the selective use of lobe-specific or tailored lymphadenectomy in carefully staged, low-risk early disease. Finally, emerging quality improvement interventions, including standardized specimen handling, operative checklists, and multidisciplinary feedback mechanisms, have demonstrated measurable improvements in guideline adherence and patient outcomes. This narrative review integrates contemporary evidence and guideline recommendations to outline a practical framework for implementing reliable, high-quality lymph node staging in modern lung cancer surgery.

## 1. Introduction

Precise lymph node staging is a cornerstone of contemporary non-small-cell lung cancer (NSCLC) management, influencing diagnosis, therapeutic strategy, and long-term prognostication. Although advances in cross-sectional imaging, positron emission tomography, and minimally invasive endoscopic techniques have improved preoperative assessment, pathological evaluation of lymph nodes at the time of surgical resection continues to represent the most reliable method for defining the true anatomic extent of disease. A substantial body of evidence demonstrates that inadequate nodal evaluation, manifested by examination of too few lymph nodes, omission of critical mediastinal stations, or failure to perform systematic nodal assessment, leads to pathologic understaging, missed occult N2 metastases, reduced use of adjuvant therapy, and inferior survival outcomes following lung cancer surgery [1,2,3,4].

In response to these observations, lymph node staging has evolved from a technical consideration into a central quality metric in thoracic oncology. The Commission on Cancer (CoC) now explicitly defines adequate nodal evaluation for curative-intent pulmonary resection as sampling of at least one N1 (hilar or intrapulmonary) station and three distinct N2 (mediastinal) stations, reflecting a shift away from simple lymph node counts toward anatomically structured, station-based assessment [5]. Concordant guidance from the National Comprehensive Cancer Network (NCCN) and the European Society of Thoracic Surgeons (ESTS) similarly emphasizes systematic evaluation of both hilar and mediastinal nodal basins, including mandatory sampling of a minimum of three mediastinal stations with inclusion of the subcarinal node to support accurate assignment of pN0 status [5,6,7]. The International Association for the Study of Lung Cancer (IASLC) reinforces this framework by defining adequacy through anatomically appropriate station clearance and underscoring the oncologic consequences of incomplete mediastinal evaluation [8].

Parallel advances in preoperative mediastinal staging have further refined surgical decision-making. Endobronchial ultrasound-guided transbronchial needle aspiration (EBUS-TBNA), often combined with esophageal ultrasound-guided fine-needle aspiration (EUS-FNA), has become the preferred first-line invasive staging modality for patients with suspicious mediastinal findings, central tumors, or radiographic N1 disease. This paradigm shift has substantially reduced reliance on routine mediastinoscopy while enabling more accurate identification of patients who may benefit from neoadjuvant therapy or alternative treatment strategies [9,10,11]. Conversely, for small, peripheral clinical T1 tumors without radiographic evidence of nodal involvement, guidelines allow omission of invasive preoperative staging, reflecting a risk-adapted approach that balances diagnostic yield against procedural burden [9,12].

The appropriate intraoperative extent of lymphadenectomy remains an area of ongoing investigation. Evidence from randomized trials and large observational studies indicates that, in rigorously staged early-stage NSCLC, particularly clinical T1–2N0 disease, systematic multi-station nodal sampling can achieve staging accuracy and survival outcomes comparable to those of complete mediastinal lymph node dissection (MLND). Nonetheless, full MLND continues to play a critical role in patients with higher-risk features or in cases where preoperative mediastinal assessment is incomplete or uncertain [13,14,15]. More recently, selective and lobe-specific lymph node dissection strategies, informed by predictable lymphatic drainage patterns, have demonstrated oncologic non-inferiority in carefully selected early-stage tumors, provided that minimum guideline-defined station requirements are fulfilled [16,17].

At the population level, the implications of nodal staging quality extend well beyond individual patient outcomes. Analyses of large national databases consistently show that more comprehensive lymphadenectomy is associated with higher rates of nodal upstaging, lower recurrence, and improved long-term survival. Moreover, hospitals and surgeons who consistently meet guideline-based nodal staging standards outperform lower-compliance counterparts across multiple quality indicators [1,2,3,4,5,18]. These observations have driven the adoption of standardized nodal evaluation protocols, specimen-collection systems, and targeted quality-improvement (QI) initiatives aimed at reducing practice variability and improving adherence to evidence-based standards [5,18].

Taken together, these converging lines of evidence underscore that adequate lymph node staging is not merely a technical aspect of lung resection but a fundamental oncologic principle with direct implications for patient survival and institutional performance. This review synthesizes contemporary data supporting optimal nodal assessment, summarizes current international recommendations, and discusses practical strategies for integrating guideline-concordant lymphadenectomy into modern lung cancer surgery and quality-improvement programs. The evidence synthesis was informed by a targeted literature search of PubMed/MEDLINE and Embase databases covering publications from January 2000 through March 2025. Search terms included combinations of “non-small-cell lung cancer,” “lymph node staging,” “mediastinal lymphadenectomy,” “nodal upstaging,” “EBUS,” “mediastinoscopy,” and “quality improvement.” Priority was given to randomized controlled trials, large population-based and registry studies, meta-analyses, and contemporary international guidelines from the NCCN, ESTS, IASLC, and Commission on Cancer. Additional relevant articles were identified through manual review of reference lists. The intent was to synthesize high-quality evidence and guideline recommendations rather than perform a systematic review or meta-analysis.

## 2. Rationale for Adequate Lymph Node Staging in Lung Cancer

Accurate assessment of lymph node involvement is central to lung cancer management, as nodal status remains the most powerful predictor of long-term survival and the primary determinant of postoperative treatment strategy. Pathologic nodal classification (pN) directly informs decisions regarding adjuvant chemotherapy, immunotherapy, and radiotherapy, and serves as a key prognostic anchor across all disease stages. Of note, even in an era characterized by advanced imaging modalities and molecular risk stratification, the presence of nodal metastases continues to fundamentally alter tumor behavior and clinical outcomes, reinforcing the necessity for precise and comprehensive nodal evaluation [19].

The biological heterogeneity of NSCLC further amplifies the importance of meticulous lymph node staging. A meaningful proportion of patients deemed node-negative on preoperative imaging are ultimately found to harbor microscopic metastatic disease within hilar or mediastinal lymph nodes. Such occult N1 or N2 involvement can only be identified through systematic lymphadenectomy or rigorous multi-station nodal sampling. Evidence from large population-based datasets, including analyses derived from the National Cancer Database (NCDB) and the Surveillance, Epidemiology, and End Results (SEER) program, consistently demonstrates that more extensive nodal evaluation, measured by both the number of examined lymph nodes (ELNs) and the breadth of station sampling, is associated with higher rates of nodal upstaging, improved staging accuracy, and superior long-term survival [1,3,4,5,18,20].

Quantitative analyses have defined clinically meaningful thresholds for adequate nodal evaluation. In a landmark population-based study including over 20,000 patients with resected T1–3N0M0 NSCLC, Liang Et Al. demonstrated that examination of at least 10 lymph nodes was associated with significantly improved overall survival compared with lower nodal yields, reflecting more accurate pathologic staging and reduced stage migration [4]. Subsequent analyses integrating the National Cancer Database and multi-institutional registries suggested that examination of up to 16 lymph nodes further optimized staging precision, with node-negative patients below this threshold experiencing inferior long-term survival consistent with occult understaging [5,6]. Across contemporary cohorts, systematic mediastinal evaluation increases detection of occult N2 disease by approximately 8–15%, depending on tumor characteristics and staging rigor, thereby directly influencing eligibility for adjuvant systemic therapy and long-term outcomes [1,3,5,18].

Conversely, insufficient lymph node assessment carries substantial oncologic consequences. Patients who are understaged due to limited nodal sampling are less likely to receive guideline-recommended adjuvant therapy, thereby forfeiting proven survival benefits. Residual, undetected nodal disease contributes to increased risks of locoregional recurrence and distant metastatic progression. Observational studies consistently show that inadequate lymphadenectomy is associated with marked survival disadvantages; in particular, patients classified as node-negative following resections in which no lymph nodes were examined experience significantly worse outcomes compared with those undergoing systematic mediastinal and hilar evaluation [1,4,18].

From the perspective of surgical oncology, lymph node staging also serves as a surrogate indicator of operative quality and oncologic completeness. Compliance with guideline-based nodal assessment has emerged as a core measure of surgical performance, analogous to achieving an R0 resection margin. In recognition of this, both the Commission on Cancer (CoC) and the National Comprehensive Cancer Network (NCCN) have incorporated explicit lymphadenectomy requirements, most notably the sampling of at least one N1 and three N2 stations, into national quality frameworks. Adherence to these standards is increasingly linked to institutional accreditation, public reporting, and performance benchmarking [5,6,14]. Centers that consistently meet these criteria demonstrate more accurate staging, improved stage-specific survival, and higher rates of appropriate postoperative therapy administration [6,14,16].

Beyond individual patient management, high-quality lymphadenectomy enhances the interpretability of surgical outcomes at the population level and strengthens the validity of comparative-effectiveness research. Institutions with poor adherence to nodal staging standards frequently exhibit distorted survival patterns, including artificially favorable outcomes in early-stage cohorts due to understaging and inferior outcomes in advanced-stage disease resulting from missed opportunities for multimodality treatment. In contrast, centers that reliably perform systematic nodal evaluation achieve more accurate “stage purification”, enabling refined prognostic stratification, improved alignment of therapy with disease burden, and more meaningful institutional and interventional comparisons [1,16,21].

Collectively, these oncologic, prognostic, and quality-related considerations establish comprehensive lymph node staging as a foundational element of modern lung cancer care. Accurate nodal evaluation not only sharpens staging precision and guides postoperative treatment but also functions as a key determinant of surgical quality, institutional performance, and ultimately patient survival.

## 3. Guideline Framework: NCCN, CoC, ESTS, and IASLC (2024–2025)

Over the past several years, international societies have progressively aligned their recommendations regarding what constitutes adequate lymph node staging in resectable NSCLC. Earlier guidance differed substantially between organizations, with some emphasizing absolute lymph node counts and others prioritizing anatomic station sampling. Since 2021, and with further clarification through 2024–2025 updates, major guideline bodies—including the NCCN, CoC, European Society of Thoracic Surgeons (ESTS), and International Association for the Study of Lung Cancer (IASLC)—have converged on a more uniform, station-based definition of nodal adequacy that prioritizes systematic mediastinal evaluation and reproducible intraoperative technique (Table 1).

Among these, the CoC Operative Standard 5.8 provides one of the most explicit and operationalized frameworks. Introduced in 2021 and subsequently reinforced through national implementation analyses, this standard defines adequate lymphadenectomy during curative-intent pulmonary resection as the assessment of at least one hilar or intrapulmonary (N1) lymph node station and a minimum of three distinct mediastinal (N2) stations [5,14,22]. The transition from node-count-based benchmarks to station-based requirements reflects accumulating evidence that comprehensive station coverage more reliably captures mediastinal disease and correlates more closely with accurate pathologic staging and survival. In this context, population-level compliance studies demonstrate that patients classified as pN0 who do not meet this standard experience inferior overall survival, strongly suggesting clinically meaningful occult understaging when nodal assessment is incomplete [2,14].

NCCN recommendations parallel this approach and emphasize systematic hilar and mediastinal evaluation as an integral component of curative lung cancer surgery. Contemporary NCCN summaries and surgeon-directed educational materials consistently reference the expectation to assess at least one N1 and three N2 stations, aligning closely with the CoC framework [6,23]. While NCCN acknowledges that invasive preoperative staging may be omitted in select low-risk scenarios, such as small, peripheral tumors less than 3 cm without radiographic nodal involvement, it continues to stress that intraoperative nodal sampling remains mandatory, even in these cases. This stance underscores the organization’s broader emphasis on anatomic completeness and the limitations of imaging alone in excluding occult nodal disease.

The ESTS guidelines offer a complementary yet more prescriptive framework that has long influenced European practice. ESTS explicitly advocates systematic lymphadenectomy during anatomic lung resection, defining adequacy as removal of lymphatic tissue from hilar, interlobar, and mediastinal stations, with mandatory sampling of at least three mediastinal stations and compulsory inclusion of the subcarinal (station 7) node [7]. In addition, ESTS recommends retrieval of at least six lymph nodes across hilar and mediastinal regions to reliably assign pN0 status. This approach, grounded in European surgical traditions and supported by multiple validation studies, continues to serve as a benchmark for surgical completeness in clinical trials, registries, and institutional audits.

Rather than focusing on numeric thresholds alone, the IASLC emphasizes anatomically appropriate nodal evaluation tailored to tumor laterality and lobe-specific lymphatic drainage patterns. IASLC staging and quality documents define adequate mediastinal assessment through clearance of nodal stations most relevant to tumor location (for example, stations 2R, 4R, and 7 for right-sided tumors, and stations 4L, 5, 6, and 7 for left-sided tumors) combined with systematic hilar evaluation [8,24]. IASLC publications further caution that incomplete mediastinal assessment may render a resection oncologically “uncertain”, with implications for R-status classification and postoperative management. Consistent with other societies, IASLC analyses also demonstrate that centers performing more thorough lymphadenectomy achieve higher rates of nodal upstaging and improved survival, reinforcing the oncologic importance of structured nodal evaluation.

Taken together, these contemporary guidelines articulate a shared principle: adequate lymph node staging requires deliberate, systematic evaluation of both hilar and mediastinal nodal basins, sampling multiple anatomically relevant stations in accordance with established drainage pathways. While minor differences persist in how individual societies operationalize these recommendations, all converge on the central importance of multi-station, station-based assessment as a defining feature of high-quality lung cancer surgery. In modern practice, the goal is no longer simply lymph node removal, but rather a reproducible, anatomically informed, and guideline-concordant lymphadenectomy that ensures accurate staging, guides postoperative therapy, and ultimately improves patient outcomes.

## 4. Preoperative Mediastinal Staging (EBUS/EUS ± Mediastinoscopy)

### 4.1. Radiologic Assessment and Indications for Invasive Staging

Accurate preoperative mediastinal staging is a critical determinant of treatment strategy in patients with potentially resectable NSCLC. Although advances in cross-sectional imaging with computed tomography and functional assessment with positron emission tomography have substantially improved baseline evaluation, neither modality provides sufficient sensitivity to reliably exclude nodal metastasis. This limitation is particularly evident in patients with central tumors, radiographic cN1 disease, or small-volume N2 involvement that may escape detection on imaging alone. Consequently, invasive mediastinal staging remains indispensable for defining operability and identifying patients who may benefit from neoadjuvant therapy prior to resection [24,25,26,27].

Over the last decade, endobronchial ultrasound-guided transbronchial needle aspiration (EBUS-TBNA), often combined with esophageal ultrasound-guided fine-needle aspiration (EUS-FNA), has emerged as the preferred first-line invasive staging modality across international guidelines. Updated ESTS recommendations, ACCP-aligned guidance, and contemporary consensus reviews uniformly endorse an endosonography-first strategy whenever the pretest probability of mediastinal metastasis exceeds a minimal threshold [24,25,26,27]. This shift reflects robust evidence demonstrating that EBUS/EUS offers high sensitivity for detecting N2 and N3 disease, excellent safety, and the ability to access a broad range of mediastinal and hilar nodal stations. Within this modern framework, endosonography has transitioned from a supplementary tool to the central pillar of invasive mediastinal staging.

Current guidelines identify several clinical scenarios in which preoperative invasive staging is strongly indicated. These include the presence of enlarged or FDG-avid mediastinal lymph nodes on CT or PET imaging, centrally located primary tumors even in the absence of radiographic nodal abnormalities, and radiographic cN1 disease, given its well-documented association with occult N2 metastasis [24,25,26,27]. In clinical practice, SUVmax thresholds in the range of 2.5–3.0 are commonly used to prompt invasive mediastinal staging, although these values should be interpreted cautiously due to the potential for false-positive uptake related to inflammation, infection, or granulomatous disease [24,25,26]. Accordingly, PET findings should be integrated with computed tomography characteristics, tumor location, histology, and overall pretest probability when determining the need for invasive nodal sampling. In addition, many recommendations extend invasive staging to patients with larger primary tumors or lesions exhibiting high metabolic activity, particularly adenocarcinomas, where the baseline likelihood of mediastinal involvement remains substantial despite negative imaging findings [25,26,27].

On computed tomography, mediastinal lymph nodes are generally considered suspicious when the short-axis diameter is ≥10 mm; however, size criteria alone have limited sensitivity, as micrometastatic disease may be present in morphologically normal nodes [24,25,26,27]. Positron emission tomography provides complementary functional assessment, with nodal fluorodeoxyglucose uptake exceeding background mediastinal activity typically regarded as abnormal. In clinical practice, SUVmax thresholds between 2.5 and 3.0 are commonly used to prompt invasive evaluation, although false-positive uptake related to inflammation or granulomatous disease must be considered [24,25,26]. Consequently, radiologic findings should be interpreted in conjunction with tumor location, histology, and overall pretest probability of mediastinal involvement.

Conversely, invasive mediastinal staging may be safely omitted in a narrowly defined subset of patients. This group includes individuals with small (≤3 cm), clearly peripheral clinical T1N0 tumors and no suspicious mediastinal nodes on CT or PET imaging [24,25,26]. Notably, guidelines uniformly stress that omission of preoperative EBUS/EUS in such low-risk cases does not equate to omission of nodal evaluation altogether. Systematic intraoperative assessment of hilar and mediastinal lymph nodes remains mandatory at the time of surgical resection to ensure accurate final pathologic staging.

### 4.2. Role of Mediastinoscopy After Negative or Inconclusive Endosonography

A long-standing area of debate has concerned the role of confirmatory mediastinoscopy following a negative EBUS/EUS examination. This question was directly addressed by the randomized MEDIASTrial, a multicenter non-inferiority study evaluating endosonography with or without confirmatory mediastinoscopy in patients with resectable NSCLC and a high pretest probability of mediastinal nodal disease [28,29]. A total of 360 patients were randomized, with 178 assigned to endosonography alone and 182 to endosonography followed by confirmatory mediastinoscopy [28]. The primary endpoint—detection of unforeseen N2 disease at surgical resection—occurred in 8.8% of patients in the endosonography-only group and 7.7% in the confirmatory mediastinoscopy group, meeting predefined criteria for non-inferiority. No significant differences were observed in overall survival or disease-free survival between strategies during follow-up [28,29]. These findings support a selective rather than routine use of mediastinoscopy following a negative, high-quality EBUS/EUS examination in appropriately staged patients. Subsequent guideline-oriented analyses and expert reviews have interpreted these findings as justification for abandoning routine confirmatory mediastinoscopy in favor of a selective, risk-adapted approach [26,27]. In contemporary practice, mediastinoscopy is therefore reserved for specific high-risk scenarios, including patients with an exceptionally high pretest probability of mediastinal disease despite negative endosonography, technically limited or incomplete EBUS/EUS examinations, discordant imaging and biopsy results, or selected restaging situations following neoadjuvant therapy [25,26,27].

When EBUS/EUS-guided sampling is negative or inconclusive, subsequent management should be individualized according to the estimated pretest probability of mediastinal disease. In patients with high-risk features, such as centrally located tumors, radiographically enlarged or FDG-avid mediastinal nodes, cN1 disease, or technically limited endosonographic sampling, surgical mediastinoscopy remains appropriate to exclude occult N2 disease prior to resection [25,26,27]. Conversely, in patients with low-risk profiles, including small peripheral tumors and comprehensive, high-quality EBUS/EUS sampling of all relevant stations, proceeding directly to surgical resection with systematic intraoperative lymph node evaluation is considered acceptable and guideline-concordant [26,27,28,29]. This risk-adapted strategy balances diagnostic accuracy with procedural burden and reflects contemporary staging algorithms.

As a result of these developments, endoscopic staging has become the cornerstone of modern mediastinal evaluation. Its widespread adoption has markedly reduced dependence on surgical mediastinoscopy, improved preoperative risk stratification, and facilitated more rational selection of patients for surgery, neoadjuvant treatment, or definitive nonsurgical management. Within this contemporary algorithm, preoperative mediastinal staging serves not only to confirm or exclude nodal metastasis but also to inform the anticipated extent of intraoperative lymphadenectomy, refine prognostic assessment, and optimize sequencing of systemic and surgical therapies [24,25,26,27].

In summary, preoperative mediastinal staging has evolved into a highly individualized, algorithm-driven process that integrates clinical features, imaging findings, and endoscopic sampling to deliver precise nodal assessment. The accuracy, safety, and clinical impact of EBUS/EUS render high-quality endosonographic staging an essential, and non-negotiable, component of guideline-concordant lung cancer care [25,26,27].

## 5. Intraoperative Nodal Assessment: Minimum Standards and Surgical Techniques

Intraoperative lymph node evaluation remains the definitive step for establishing pathologic stage in NSCLC and represents a cornerstone of curative lung cancer surgery. While imaging and endoscopic techniques have substantially improved preoperative risk stratification, only surgical lymphadenectomy allows for complete assessment of nodal involvement, identification of micrometastatic disease, and assignment of an accurate postoperative pN category. Reflecting this central role, contemporary guidance from NCCN, the CoC, ESTS, and IASLC-aligned reviews has shifted emphasis away from simple lymph node counts toward structured, station-based intraoperative assessment grounded in anatomic and oncologic principles [1,2,7,9,20,24].

The most widely implemented minimum standard derives from the CoC Operative Standard 5.8 and its alignment with NCCN expectations. This framework defines adequacy by the removal or sampling of lymph nodes from at least one N1 (hilar or intrapulmonary) station and at least three distinct N2 (mediastinal) stations during any curative-intent pulmonary resection [7,8,9,10]. This transition from numeric node thresholds to station-based criteria reflects evidence that node counts alone can be misleading, as nodal yield varies substantially by anatomic station and surgical approach. Analyses of national datasets consistently demonstrate that adherence to this station-based standard is associated with superior staging accuracy, higher rates of nodal upstaging, and improved long-term outcomes in patients classified as pN0, whereas failure to meet these criteria is linked to occult understaging and inferior survival [7,8].

European practice patterns, codified in ESTS guidelines, provide a complementary but more prescriptive definition of systematic lymphadenectomy. ESTS recommends formal removal of lymphatic tissue from hilar, interlobar, and mediastinal regions, with mandatory sampling of at least three mediastinal stations and obligatory inclusion of the subcarinal (station 7) node [2]. To support reliable designation of pN0 status, ESTS further advises removal of a minimum of six lymph nodes across mediastinal and hilar stations. These recommendations, derived from European surgical experience and validated in multiple clinical studies, continue to serve as influential benchmarks for operative completeness and trial design [2,3,14].

Rather than defining adequacy through numeric thresholds, the IASLC emphasizes anatomically appropriate lymphadenectomy tailored to tumor laterality and known patterns of lymphatic drainage. In this model, right-sided tumors typically require assessment of stations 2R, 4R, and 7, whereas left-sided tumors necessitate evaluation of stations 4L, 5, 6, and 7, in addition to systematic hilar sampling [1,11,20]. IASLC-aligned analyses underscore that omission of anatomically relevant mediastinal stations constitutes an oncologic compromise and may render a resection “uncertain” from a staging perspective, complicating postoperative decision-making regarding adjuvant therapy [1,11]. For practical implementation, recommended mediastinal lymph node stations according to tumor lobe and laterality are summarized in Table 2, integrating IASLC anatomic principles with contemporary CoC, NCCN, and ESTS requirements.

From a technical standpoint, intraoperative nodal assessment may be performed using several approaches, including systematic sampling, systematic nodal dissection (often termed MLND), or lobe-specific lymph node dissection. Systematic sampling involves targeted removal of representative nodes from all required stations, whereas systematic dissection entails complete clearance of nodal tissue within those stations. Randomized trials and observational studies indicate that when multi-station sampling is performed rigorously and according to predefined standards, oncologic outcomes in well-staged early disease, particularly clinical T1–2N0 tumors, approximate those achieved with complete mediastinal dissection [3,12,13,14]. In contrast, patients with higher-risk features, limited or incomplete preoperative staging, central tumors, or suspected N1 involvement continue to benefit from full systematic dissection to ensure accurate pathologic staging and optimal oncologic control [3,12,20].

Building on these principles, lobe-specific and selective lymphadenectomy strategies have gained acceptance for carefully selected early-stage NSCLC. Propensity-matched studies, systematic reviews, and prospective trial protocols demonstrate that lobe-specific dissection can achieve staging accuracy and survival outcomes comparable to systematic MLND in clinical stage I–II disease, while offering potential reductions in operative time, blood loss, and surgical morbidity [16,17,18,19]. Importantly, these approaches do not abandon systematic evaluation but instead refine it, maintaining multi-station N1 and N2 assessment in accordance with CoC and ESTS minimums. As such, lobe-specific dissection should be viewed as an anatomically informed adaptation for low-risk, rigorously staged patients rather than a lesser form of oncologic surgery [15,16,17,18,19,20].

The technical execution of lymphadenectomy varies by surgical approach (open thoracotomy, video-assisted thoracoscopic surgery (VATS), or robotic-assisted thoracic surgery (RATS)), yet fundamental oncologic principles remain unchanged. Adequate exposure of the mediastinum, precise identification of nodal stations, and meticulous dissection that balances completeness with preservation of critical structures are essential regardless of technique. Contemporary series demonstrate that experienced minimally invasive surgeons can achieve nodal yields and station coverage equivalent to open surgery, confirming that VATS and robotic platforms are fully compatible with guideline-concordant lymphadenectomy when performed in high-volume settings [20,21].

Equally critical to intraoperative quality is appropriate specimen handling. Combining lymph nodes from different stations or inaccurate labeling undermines staging precision and compromises multidisciplinary decision-making. Standardized lymph node specimen-collection kits, developed through IASLC-linked quality initiatives and evaluated in prospective and registry-based studies, have been shown to improve adherence to station-based standards, reduce the proportion of resections classified as “R-uncertain”, and enhance long-term survival in cohorts previously affected by understaging [1,22,23]. By operationalizing guideline requirements at the point of care, these tools transform lymphadenectomy from an individual surgeon-dependent practice into a reproducible, system-level process.

In summary, intraoperative lymph node assessment is not a technical afterthought but a fundamental oncologic obligation. Consistent adherence to guideline-defined minimum standards, respect for anatomic lymphatic pathways, and meticulous surgical technique are essential for accurate staging and appropriate postoperative management. As surgical strategies continue to evolve toward minimally invasive and lobe-specific approaches, the commitment to systematic, anatomically informed nodal evaluation must remain central to high-quality lung cancer surgery and quality-improvement efforts [1,2,3,7,8,9,12,14,16,17,18,19,20,22,23].

## 6. Extent of Lymphadenectomy: MLND Versus Systematic Sampling and Lobe-Specific Approaches

### 6.1. Systematic Sampling Versus Complete Mediastinal Lymph Node Dissection

The appropriate extent of lymphadenectomy in NSCLC has long been debated and continues to evolve alongside advances in staging accuracy, surgical technique, and perioperative management. For many years, complete mediastinal lymph node dissection (MLND) was regarded as the default oncologic standard for all resectable tumors, based on the premise that more extensive nodal clearance would translate into improved survival. However, accumulating evidence from randomized trials, meta-analyses, and population-based studies has demonstrated that the oncologic value of MLND is strongly dependent on disease stage and the quality of preoperative and intraoperative staging [3,12,13,14,20]. With the widespread adoption of PET/CT and endosonographic mediastinal staging, a more individualized, risk-adapted paradigm has emerged.

The American College of Surgeons Oncology Group Z0030 trial remains the most influential study informing this shift. In this randomized trial, patients with rigorously staged clinical T1–T2N0 NSCLC underwent either complete MLND or systematic multi-station mediastinal sampling after confirmation of negative nodes at predefined stations [13]. Importantly, the ACOSOG Z0030 trial mandated a meticulous, pre-defined mediastinal sampling protocol before randomization, including evaluation of laterality-specific stations (2R, 4R, and 7 for right-sided tumors; 4L, 5, 6, and 7 for left-sided tumors) [13]. Among 1111 randomized patients, 1023 were eligible and evaluable (498 assigned to systematic sampling and 525 to complete mediastinal lymph node dissection) [13]. At a median follow-up of 6.5 years, there were no significant differences in overall survival, disease-free survival, or locoregional recurrence between groups; median survival was 8.1 years with systematic sampling versus 8.5 years with complete dissection (*p* = 0.25), and 5-year disease-free survival was 69% versus 68%, respectively (*p* = 0.92) [13]. Occult N2 disease was identified in 21 patients in the complete dissection arm (≈4%), underscoring that when rigorous multi-station sampling is performed, complete dissection does not provide additional survival benefit in carefully staged early-stage NSCLC [12,13,14].

Subsequent analyses in the modern staging era have reinforced these findings. Meta-analyses integrating earlier randomized trials with contemporary observational cohorts consistently demonstrate that MLND does not provide a reproducible survival advantage over systematic sampling in patients with radiographically and endoscopically staged N0 disease, provided that sampling includes an adequate number of appropriately distributed mediastinal stations [3,12,14]. Apparent survival differences reported in older studies are now understood to largely reflect inadequate preoperative staging or incomplete nodal assessment in the sampling arms rather than an inherent therapeutic superiority of MLND [3,12,14].

### 6.2. Lobe-Specific and Selective Lymph Node Dissection

Building on this foundation, lobe-specific lymph node dissection (L-SLND) has emerged as a further refinement of surgical strategy in selected patients. L-SLND is predicated on the predictable patterns of lymphatic drainage associated with each pulmonary lobe, allowing targeted clearance of nodal stations most likely to harbor metastases while avoiding unnecessary dissection of unrelated mediastinal regions. Early feasibility studies established the safety of this approach [15,16], and more recent prospective protocols and trials, including the LobE-Specific lymph node diSsectiON trial, suggest that lobe-specific dissection may achieve oncologic outcomes comparable to systematic MLND in carefully selected clinical T1–T2N0 populations [17,18,19]. Retrospective series and registry-based analyses further demonstrate similar survival, nodal upstaging, and recurrence patterns when L-SLND is applied to low-risk tumors with thorough preoperative staging [16,17,18,19]. It is important to emphasize that lobe-specific lymph node dissection should not be interpreted as a universal alternative to systematic mediastinal lymphadenectomy. Its application is appropriately restricted to carefully selected patients with rigorously staged, low-risk early-stage NSCLC, typically characterized by small (≤3 cm), peripheral tumors, negative preoperative PET/CT, and comprehensive negative endosonographic staging when indicated. Patients with centrally located tumors, radiographic or metabolic suspicion of nodal disease, cN1 involvement, incomplete preoperative mediastinal assessment, or high-risk histologic features should continue to undergo systematic mediastinal lymph node dissection to ensure accurate staging and oncologic completeness. Framing lobe-specific dissection within this risk-adapted context is essential to avoid understaging and inappropriate extrapolation to higher-risk populations.

An additional advantage of lobe-specific strategies is their potential to reduce operative time, blood loss, and postoperative morbidity, considerations that are particularly relevant in the era of minimally invasive surgery [17,18,19,20]. Nonetheless, these benefits must be balanced against the risk of unrecognized nodal disease outside expected drainage pathways. This concern is especially pertinent in adenocarcinomas with aggressive histologic features, high metabolic activity on PET imaging, or radiographic characteristics suggestive of atypical spread, where reliance on lobe-specific patterns alone may underestimate true mediastinal involvement [3,15,20,24].

### 6.3. Morbidity Considerations and Technical Factors

Although systematic mediastinal lymph node dissection is generally safe in experienced hands, it is associated with a small but measurable increase in procedure-specific morbidity. Reported complications include chylothorax, recurrent laryngeal nerve injury, and bronchial or vascular injury, with overall incidence rates typically ranging from 1% to 5% depending on patient factors, surgical approach, and extent of dissection [12,14,20]. Chylothorax remains the most characteristic lymphadenectomy-related complication, occurring in approximately 0.5–2% of lung resections and more frequently associated with right-sided upper mediastinal dissection involving stations 2R and 4R, particularly when energy devices are used near the thoracic duct or its tributaries [14,20]. Left-sided dissections may also carry a risk of recurrent laryngeal nerve injury, especially during clearance of stations 4L and 5. Of note, contemporary series demonstrate that when nodal dissection is performed in a systematic, anatomically informed manner, these complications remain uncommon and should be weighed against the oncologic consequences of inadequate staging.

Accordingly, complete MLND continues to play a critical role in patients with higher-risk disease. Indications favoring full dissection include radiographically suspicious mediastinal nodes, centrally located tumors, cN1 disease, incomplete or limited preoperative staging, tumors exceeding 3 cm with high FDG uptake, and any intraoperative finding, such as positive frozen section or gross nodal abnormality, that raises concern for mediastinal metastasis [3,12,14,20,24,25,26,27]. In these contexts, MLND enhances staging accuracy and may provide therapeutic benefit by removing occult micrometastatic disease not reliably captured by limited sampling.

Across these diverse strategies, a consistent theme emerges: the quality and completeness of nodal assessment are more important than the nominal extent of dissection. When surgeons adhere to guideline-defined standards, particularly adequate station coverage with at least one N1 and three N2 stations, and apply anatomically appropriate techniques, systematic sampling, systematic dissection, and lobe-specific approaches yield comparable oncologic outcomes in early, low-risk NSCLC [1,3,6,7,12,13,14,15,16,18,19,20]. In contrast, inadequate sampling, regardless of whether it occurs during an ostensibly “complete” or “selective” operation, is consistently associated with inferior staging accuracy, underestimated recurrence risk, and worse survival [1,4,5,6,7,16,18,23].

In contemporary practice, lymphadenectomy is therefore best conceptualized as a risk-adapted intervention rather than a uniform requirement. For rigorously staged early-stage NSCLC, lobe-specific or systematic sampling strategies that meet established minimum standards offer oncologically sound and potentially less morbid alternatives to MLND. Conversely, in higher-risk disease or when staging uncertainty persists, complete mediastinal dissection remains indispensable. Across all scenarios, the overarching objective is not maximal tissue removal, but high-quality, anatomically informed nodal assessment that ensures accurate staging and optimizes long-term oncologic outcomes.

## 7. Impact of Inadequate Staging on Survival, Recurrence, Upstaging, and Quality Metrics

**Figure 1:**
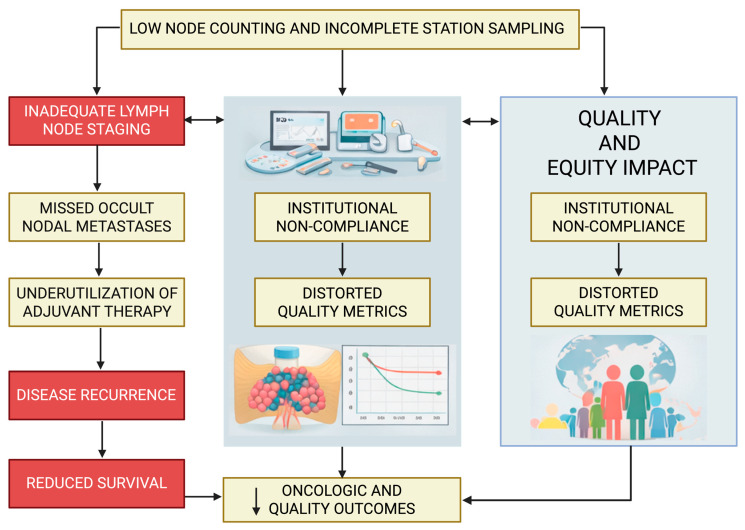
Conceptual framework illustrating the downstream clinical, institutional, and equity-related consequences of inadequate lymph node staging in non-small-cell lung cancer. Low lymph node counts and incomplete station sampling lead to inadequate nodal staging, resulting in missed occult nodal metastases, underutilization of adjuvant therapy, increased recurrence, and reduced survival. At the institutional level, inadequate staging contributes to non-compliance with guideline-defined quality standards, distorted quality metrics, and impaired benchmarking. These effects collectively translate into poorer oncologic outcomes and exacerbate disparities in quality and equity of lung cancer care.

While Section 2 outlines the biologic and oncologic rationale for comprehensive lymph node staging, this section focuses specifically on the downstream consequences of inadequate staging, including survival, recurrence, nodal upstaging, and performance against established quality metrics. The ramifications of inadequate lymph node staging extend well beyond imprecision in pathologic classification and have profound consequences for oncologic outcomes, staging integrity, and health-system performance. Across large national datasets, multi-institutional registries, and guideline-driven analyses, insufficient lymphadenectomy—defined by limited nodal yield, inadequate mediastinal station coverage, or omission of systematic assessment—has been consistently linked to inferior survival, higher recurrence rates, reduced detection of occult nodal disease, and poorer adherence to established quality benchmarks [1,2,4,5,6,7,8,11,12,16,18,22,23] (Table 3). These interconnected downstream effects are summarized schematically in Figure 1, which illustrates how inadequate lymph node evaluation leads to occult nodal disease, inappropriate omission of adjuvant therapy, increased recurrence, distorted quality metrics, and ultimately inferior survival at both the patient and institutional levels.

One of the most consistent and reproducible observations in lung cancer outcomes research is the association between the extent of lymph node examination and overall survival. Analyses derived from the National Cancer Database and the Surveillance, Epidemiology, and End Results (SEER) program demonstrate a clear dose–response relationship between the extent of lymph node evaluation and survival. In node-negative patients, 5-year overall survival improves progressively as the number of examined lymph nodes increases, with reported absolute survival differences of 5–10% between patients with no or minimal nodal assessment and those undergoing systematic multi-station evaluation [3,4,5,18]. Patients classified as pN0 after resection in which no lymph nodes were examined experience survival outcomes comparable to understaged N1 disease, highlighting the oncologic consequences of inadequate nodal assessment [1,4,18]. A landmark analysis identified approximately 10 examined lymph nodes as a meaningful threshold for patients with T1–3N0M0 NSCLC, showing significant survival differences between patients with at least 10 nodes evaluated and those below this threshold [4]. Subsequent combined registry studies proposed an even higher benchmark of 16 examined nodes to optimize staging precision and long-term outcomes, observing that node-negative patients with fewer than 16 examined nodes had inferior survival and were likely understaged [5]. Collectively, these data underscore that inadequate lymphadenectomy obscures true disease burden and compromises both prognostication and therapeutic decision-making. Representative nodal upstaging rates reported in landmark randomized trials and large population-based studies are summarized in Table 4, highlighting the consistent association between comprehensive lymph node evaluation and improved staging accuracy.

The impact of insufficient nodal assessment is equally evident in patterns of recurrence. Limited sampling diminishes the likelihood of identifying N2 disease, leading to stage migration that underestimates tumor aggressiveness and results in undertreatment. Patients classified as pN0 following inadequate nodal evaluation frequently experience early locoregional or distant recurrence, suggesting that residual microscopic disease remained undetected at the time of surgery [1,4,12]. In contrast, institutions that routinely perform systematic lymphadenectomy report higher rates of nodal upstaging, enabling the appropriate use of adjuvant systemic therapy—interventions that have been shown to improve disease-free and overall survival [3,6,7,8,16].

These oncologic consequences intersect directly with quality-of-care metrics. The Commission on Cancer Operative Standard 5.8, which mandates sampling of at least one N1 and three N2 stations, was specifically designed to quantify operative thoroughness and reduce national variation in nodal staging practices [7,9,10]. Analyses examining early compliance with this standard demonstrate that while recurrence rates may appear similar at a population level, patients classified as pN0 who do not meet the standard experience significantly worse survival, thus strongly implicating occult understaging and missed opportunities for adjuvant therapy [2,7]. Importantly, centers with higher compliance rates achieve more accurate staging and improved outcomes across multiple disease stages [7,8,10].

Quality-improvement initiatives aligned with NCCN principles further illustrate the system-level impact of nodal adequacy. The introduction of standardized lymph node specimen-collection kits—designed to enforce anatomic labeling and comprehensive station submission—has been shown to substantially reduce the proportion of resections categorized as “R-uncertain.” Data from multi-institutional quality-improvement programs indicate that improved nodal submission accuracy not only enhances pathologic staging but also translates into meaningful survival gains among patients who would otherwise have been misclassified due to incomplete nodal evaluation [1,22,23]. These findings highlight that lymph node staging quality is influenced not only by surgical technique but also by coordinated operative and pathology workflows.

At the level of individual surgeons and institutions, lymphadenectomy adequacy has emerged as a robust surrogate for overall oncologic quality. Registry-based studies consistently demonstrate that surgeons and centers achieving higher nodal yields and more complete station coverage exhibit superior staging accuracy, higher upstaging rates, and improved long-term survival compared with lower-performing counterparts [1,7,11,16,23]. These patterns emphasize that lymph node assessment is not merely a technical step within the operation but a defining feature of high-performance thoracic surgery programs.

Taken together, the evidence makes clear that inadequate lymph node staging undermines nearly every dimension of lung cancer care. It compromises staging accuracy, impedes the appropriate application of multimodality therapy, increases the risk of recurrence, and ultimately worsens survival. At a systems level, insufficient staging distorts quality metrics, weakens institutional benchmarking, and contributes to persistent disparities in care delivery. Ensuring thorough, guideline-concordant lymphadenectomy therefore represents one of the most impactful and measurable quality-improvement opportunities in contemporary thoracic oncology.

## 8. Quality Improvement Implications and Institutional Strategies

### 8.1. Variability in Practice and the Need for Standardization

**Figure 2:**
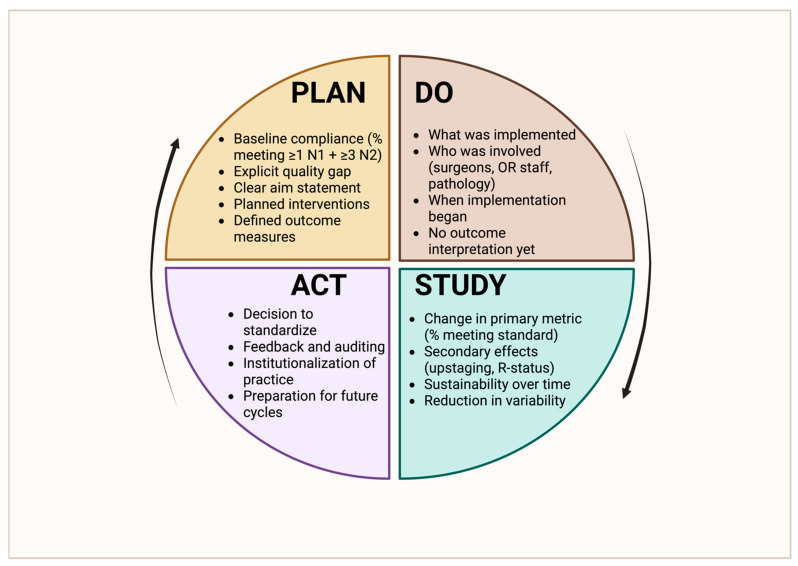
Application of the Plan–Do–Study–Act (PDSA) quality-improvement cycle to lymph node staging in non-small-cell lung cancer surgery. The framework outlines identification of baseline compliance gaps (Plan), implementation of standardized nodal staging interventions (Do), evaluation of staging adequacy and secondary outcomes such as upstaging and R-status (Study), and institutionalization of effective practices with ongoing audit and feedback (Act). This iterative approach supports sustained adherence to guideline-concordant nodal evaluation.

Building on the outcome implications discussed in Section 7, this section shifts focus from consequences to solutions, outlining institutional and system-level quality improvement strategies aimed at improving lymph node staging reliability and guideline adherence. The strong and reproducible association between comprehensive lymph node staging and improved oncologic outcomes has established lymphadenectomy adequacy as one of the most impactful and modifiable quality targets in thoracic surgery. Because insufficient nodal evaluation leads to understaging and omission of appropriate adjuvant therapy, contemporary thoracic oncology increasingly recognizes lymph node staging not as a technical detail of the operation but as a core quality indicator that reflects institutional standards, operative discipline, and multidisciplinary integration [1,5,7,9,14,16,22,23]. A structured, iterative framework for addressing these deficiencies using a Plan–Do–Study–Act (PDSA) methodology is illustrated in Figure 2, highlighting how standardized workflows, audit, and feedback can be operationalized to improve lymph node staging quality at the institutional level.

A major obstacle to achieving consistently high-quality nodal staging is the substantial variability in practice across institutions, surgeons, and healthcare systems. National compliance analyses reveal that a considerable proportion of lung cancer resections fail to meet minimum nodal standards, such as the Commission on Cancer requirement to sample at least one N1 and three N2 stations. These deficiencies are most pronounced in community hospitals and lower-volume centers, where resource constraints, workflow variability, and differences in surgical culture may limit adherence [2,7,8,9,10]. Of note, such variation is not merely procedural; it translates into measurable disparities in staging accuracy, survival, recurrence, and access to postoperative therapy, underscoring the need for systematic and scalable quality-improvement (QI) strategies.

### 8.2. Operative and Pathology-Based Interventions

One of the most effective and widely studied interventions to improve nodal staging quality is the implementation of standardized lymph node specimen-collection kits. These kits enforce anatomically precise labeling and comprehensive submission of nodal stations and have been shown to significantly increase nodal yield, improve compliance with CoC and NCCN standards, and reduce the frequency of resections classified as “R-uncertain.” Evidence from prospective and multi-institutional QI initiatives further suggests that the use of specimen-collection kits is associated with improved long-term survival among patients who would otherwise have been understaged due to incomplete nodal evaluation [1,22,23]. By converting an operator-dependent task into a structured and reproducible process, these tools align surgical practice with quality standards in a practical and sustainable manner.

Beyond physical tools, procedural standardization within the operating room represents an important lever for quality improvement. The incorporation of lymphadenectomy-specific prompts into surgical checklists, standardized operative pathways, and intraoperative time-outs focused on oncologic completeness can reduce variability between surgeons and reinforce expectations for station-based nodal assessment [1,5,16,20,24]. These approaches leverage established safety and quality frameworks and embed lymph node staging into routine operative culture, rather than treating it as an optional or secondary consideration.

Multidisciplinary collaboration is another critical determinant of staging quality. Effective coordination among surgeons, pulmonologists, radiologists, and pathologists ensures accurate preoperative risk stratification, appropriate utilization of EBUS/EUS, and meticulous handling and reporting of surgical specimens [24,25,26,27]. Multidisciplinary tumor boards provide a forum to identify patients at increased risk for inadequate staging, such as those with large or centrally located tumors or incomplete preoperative evaluation, and to proactively reinforce the need for enhanced intraoperative assessment. Postoperative pathology review, including systematic evaluation of nodal station submission and feedback to the surgical team, further strengthens accountability and reinforces quality expectations [1,22,23].

### 8.3. Audit, Feedback, and Performance Benchmarking

Data-driven auditing and performance feedback are equally essential components of institutional improvement. Programs overseen by the Commission on Cancer and regional or national registries increasingly incorporate nodal adequacy metrics into quality dashboards used for benchmarking at both the surgeon and institutional level [7,8,9,10,16,23]. Evidence from national audits indicates that transparent reporting and structured feedback, particularly when paired with clearly defined standards, lead to sustained improvements in compliance, higher rates of nodal upstaging, and improved survival among early-stage patients [1,7,16,22,23]. Integrating these metrics into morbidity and mortality conferences, quality committees, and annual performance evaluations fosters continuous refinement of surgical practice.

### 8.4. Education, Training, and Equity Considerations

Education and training represent the final cornerstone of durable improvement in lymphadenectomy quality. Simulation-based instruction, dedicated curricula for minimally invasive mediastinal lymphadenectomy, and structured mentorship or proctoring programs help ensure that both trainees and early-career surgeons acquire the technical proficiency and anatomic understanding required for high-quality nodal dissection [20,21]. Institutions that invest in formalized training pathways demonstrate greater consistency in nodal yields and station coverage across surgeons, with reduced variability and improved adherence to guideline expectations [1,16,21,23].

Beyond operative and institutional metrics, emerging evidence underscores that lymph node staging quality should be interpreted within a broader oncologic and systems-based framework. Recent contemporary analyses emphasize that adequate lymphadenectomy is not only a determinant of pathologic accuracy but also a critical enabler of equitable access to guideline-directed multimodality therapy, particularly in health systems with heterogeneous resources and variable adherence to standards [30,31]. These studies highlight that disparities in nodal assessment contribute to downstream inequities in staging, treatment allocation, and survival, reinforcing lymph node evaluation as both a technical and a health-system quality indicator. Framing lymphadenectomy adequacy within this wider context further supports its role as a cornerstone metric for quality improvement initiatives aimed at standardizing lung cancer care and improving outcomes across diverse practice environments [30,31].

Taken together, these strategies highlight that excellence in lymph node staging is inherently multidisciplinary, systematic, and data-driven. Achieving consistent, guideline-concordant lymphadenectomy requires more than individual technical expertise; it demands institutional commitment, clearly articulated standards, structured tools, coordinated teamwork, and ongoing performance evaluation. As lung cancer care becomes increasingly complex and personalized, rigorous nodal staging remains a foundational element of surgical quality and a critical determinant of patient outcomes. Quality-improvement initiatives that promote reliable and reproducible lymphadenectomy therefore represent a powerful opportunity to improve survival and reduce unwarranted variation across thoracic surgical programs.

## 9. Conclusions

Accurate lymph node staging remains a cornerstone of high-quality surgical care for patients with non-small-cell lung cancer and continues to exert a profound influence on oncologic outcomes. Despite substantial advances in imaging technologies, molecular profiling, and minimally invasive surgical techniques, definitive pathologic assessment of hilar and mediastinal lymph nodes remains essential for precise staging, prognostication, and postoperative treatment planning. The cumulative evidence drawn from randomized clinical trials, meta-analyses, large population-based registries, and contemporary guideline evaluations consistently demonstrates that patients who undergo thorough, guideline-concordant nodal evaluation achieve superior survival, more reliable stage classification, and the more appropriate utilization of adjuvant therapies compared with those whose nodal assessment is incomplete.

Modern data indicate that the value of lymphadenectomy lies not in indiscriminate tissue removal, but in the quality, anatomic precision, and consistency of nodal evaluation. Station-based standards, risk-adapted surgical strategies, and structured intraoperative workflows have shifted the focus from the extent of dissection alone to the reliability of staging achieved. When applied rigorously, systematic sampling, systematic dissection, and lobe-specific approaches can all meet oncologic objectives in appropriately selected patients, provided that established minimum standards are respected. From a broader perspective, lymph node staging has emerged as a defining metric of surgical quality, reflecting not only technical proficiency but also institutional culture, multidisciplinary coordination, and adherence to evidence-based care pathways. Efforts to standardize nodal assessment through quality-improvement initiatives, specimen-collection tools, and performance feedback mechanisms have demonstrated tangible benefits in staging accuracy and patient outcomes.

As lung cancer management becomes increasingly personalized and complex, the fundamental role of rigorous lymph node staging remains unchanged. Ensuring consistent, high-quality, guideline-concordant lymphadenectomy represents one of the most effective and scalable opportunities to improve outcomes, reduce unwarranted variation in care, and elevate the overall standard of surgical treatment for NSCLC across diverse practice environments.

## Figures and Tables

**Table 1:** Contemporary international recommendations for adequate lymph node staging during curative-intent resection for non-small-cell lung cancer.

Guideline Body	Core Principle	Minimum Intraoperative Nodal Requirements	Conceptual Emphasis
Commission on Cancer (CoC)	Station-based adequacy	≥1 N1 station and ≥3 distinct N2 stations	Replaces node-count metrics with reproducible, station-based quality benchmarks
National Comprehensive Cancer Network (NCCN)	Systematic nodal evaluation	Sampling or dissection of hilar and multiple mediastinal stations	Ensures accurate pathologic staging regardless of preoperative imaging
European Society of Thoracic Surgeons (ESTS)	Systematic lymphadenectomy	Hilar/interlobar nodes plus ≥3 mediastinal stations including station 7	Minimizes occult N2 disease and defines surgical completeness
International Association for the Study of Lung Cancer (IASLC)	Anatomically tailored staging	Laterality-specific mediastinal station assessment	Aligns nodal evaluation with biologic lymphatic drainage

Abbreviations: CoC; Commission on Cancer; NCCN; National Comprehensive Cancer Network; ESTS; European Society of Thoracic Surgeons; IASLC; International Association for the Study of Lung Cancer; NSCLC; non-small-cell lung cancer.

**Table 2:** Recommended mediastinal lymph node stations for systematic intraoperative staging according to tumor lobe.

Tumor Lobe	Mandatory Mediastinal (N2) Stations	Additional Stations Frequently Assessed	Key Guideline Sources
Right upper lobe	2R, 4R, 7	10R, 11R	CoC; NCCN; ESTS; IASLC
Right middle lobe	2R, 4R, 7	10R, 11R	CoC; NCCN; ESTS; IASLC
Right lower lobe	4R, 7, 8, 9	10R, 11R	CoC; ESTS; IASLC
Left upper lobe	4L, 5, 6, 7	10L, 11L	CoC; NCCN; ESTS; IASLC
Left lower lobe	7, 8, 9	4L, 10L, 11L	CoC; ESTS; IASLC

Footnote: Systematic nodal evaluation requires assessment of at least one N1 station and a minimum of three distinct N2 stations, in accordance with Commission on Cancer Operative Standard 5.8 and concordant NCCN and ESTS recommendations. Stations listed reflect dominant lymphatic drainage pathways and should be interpreted within a risk-adapted staging strategy rather than as an exclusive template. Abbreviations: CoC, Commission on Cancer; NCCN, National Comprehensive Cancer Network; ESTS, European Society of Thoracic Surgeons; IASLC, International Association for the Study of Lung Cancer; N1, hilar and intrapulmonary lymph node stations; N2, mediastinal lymph node stations.

**Table 3:** Oncologic and quality consequences associated with inadequate lymph node staging in non-small-cell lung cancer.

Domain	Effect of Inadequate Nodal Assessment	Supporting Evidence Trends
Overall survival	Reduced long-term survival in pN0-labeled patients	Survival decreases with fewer examined nodes and incomplete station sampling
Disease recurrence	Higher locoregional and distant recurrence rates	Reflects missed occult nodal metastases
Pathologic upstaging	Lower detection of N1/N2 disease	Leads to undertreatment and omission of adjuvant therapy
Adjuvant therapy delivery	Reduced chemotherapy or immunotherapy use	Eligibility obscured by inadequate staging
R-status classification	Increased R-uncertain resections	Linked to poor specimen labeling
Institutional quality metrics	Failure to meet guideline benchmarks	Associated with inferior registry performance

**Abbreviations:** NSCLC; non-small-cell lung cancer; pN; pathologic nodal stage; R-status; residual tumor classification.

**Table 4:** Nodal upstaging rates associated with the extent of lymph node staging in major trials and population-based studies.

Study/Dataset	Study Type	Patient Population	Extent of Nodal Evaluation	Reported Nodal Upstaging
ACOSOG Z0030 [13]	Randomized trial	Clinical T1–2N0 NSCLC	Systematic sampling vs. MLND	Occult N2 detected in ~4% with MLND
SEER registry (Liang et al.) [4]	Population-based	T1–3N0M0 NSCLC	Increasing lymph node count	Higher nodal upstaging with ≥10 examined nodes
NCDB analyses [5,6]	Population-based	Early-stage NSCLC	Node count and station-based sampling	Upstaging optimized with ≥16 examined nodes
ESTS cohort analyses [2,7]	Multicenter observational	Resected NSCLC	Systematic mediastinal staging	N2 upstaging approximately 8–15%
CoC Standard 5.8 compliance studies [7,9,10]	Registry-based	Clinical stage I NSCLC	≥1 N1 and ≥3 N2 stations sampled	Higher N1/N2 detection vs. non-compliant resections

Footnote: Reported upstaging rates vary according to tumor characteristics, preoperative staging rigor, and definitions of nodal adequacy. Across studies, more comprehensive nodal evaluation consistently increases detection of occult N1 and N2 disease, enabling appropriate adjuvant therapy and improved survival. Abbreviations: MLND, mediastinal lymph node dissection; SEER, Surveillance, Epidemiology, and End Results; NCDB, National Cancer Database; ESTS, European Society of Thoracic Surgeons; CoC, Commission on Cancer; NSCLC, non-small-cell lung cancer; N1, hilar and intrapulmonary lymph nodes; N2, mediastinal lymph nodes.

## Data Availability

The data that support the findings of this study are available from the corresponding author, upon reasonable request.
